# Integral membrane protein structure determination using pseudocontact shifts

**DOI:** 10.1007/s10858-015-9899-6

**Published:** 2015-01-22

**Authors:** Duncan J. Crick, Jue X. Wang, Bim Graham, James D. Swarbrick, Helen R. Mott, Daniel Nietlispach

**Affiliations:** 1 Department of Biochemistry, University of Cambridge, Cambridge, UK; 2 Monash Institute of Pharmaceutical Sciences, Monash University, Melbourne, Australia

**Keywords:** Pseudocontact shift, Lanthanide tag, Structure determination, Membrane proteins

## Abstract

Obtaining enough experimental restraints can be a limiting factor in the NMR structure determination of larger proteins. This is particularly the case for large assemblies such as membrane proteins that have been solubilized in a membrane-mimicking environment. Whilst in such cases extensive deuteration strategies are regularly utilised with the aim to improve the spectral quality, these schemes often limit the number of NOEs obtainable, making complementary strategies highly beneficial for successful structure elucidation. Recently, lanthanide-induced pseudocontact shifts (PCSs) have been established as a structural tool for globular proteins. Here, we demonstrate that a PCS-based approach can be successfully applied for the structure determination of integral membrane proteins. Using the 7TM α-helical microbial receptor pSRII, we show that PCS-derived restraints from lanthanide binding tags attached to four different positions of the protein facilitate the backbone structure determination when combined with a limited set of NOEs. In contrast, the same set of NOEs fails to determine the correct 3D fold. The latter situation is frequently encountered in polytopical α-helical membrane proteins and a PCS approach is thus suitable even for this particularly challenging class of membrane proteins. The ease of measuring PCSs makes this an attractive route for structure determination of large membrane proteins in general.

**Electronic supplementary material:**

The online version of this article (doi:10.1007/s10858-015-9899-6) contains supplementary material, which is available to authorized users.

## Introduction

Protein structure determination by solution-state NMR spectroscopy typically relies to a substantial extent on NOE distance information (Wüthrich 1989). However, for large proteins obtaining a sufficient number of experimental NOE restraints to form an adequate inter-proton network can be a substantial challenge that is difficult to overcome. For integral membrane proteins the large size of the protein-surfactant complex generally necessitates the use of protein perdeuteration to provide adequate spectral quality, but this severely reduces the number of protons between which NOEs can be measured, and so limits the amount of structural information available. This is a particular problem for polytopical α-helical membrane proteins where, in terms of tertiary structure, the most important NOEs arise between the non-exchangeable side chain protons on spatially proximal helices, with dipolar interactions between exchangeable backbone amide protons generally insufficient for reliable helix packing (Nietlispach et al. 2011). Selective protonation of particular groups within a perdeuterated sample, such as the selective methyl-protonation strategies pioneered by Kay and coworkers (Tugarinov et al. 2006) and subsequently extended to further methyl-containing amino acids by others (Ayala et al. 2009; Godoy-Ruiz et al. 2010), allow additional NOEs to be measured. However, such approaches may not entirely overcome the existing problems, may not be successful for all proteins and can be extremely expensive. NMR structure determination of large helical membrane proteins therefore presents a major challenge.

Seven-transmembrane helix proteins (7TMs) are a large group of proteins that include e.g. microbial rhodopsins and G-protein-coupled receptors. Work in our laboratory previously solved the structure of the 7TM phototactic receptor sensory rhodopsin II (pSRII) from *Natronomonas pharaonis* using an NOE-based approach (Gautier et al. 2010). As well as NOEs measured on ILV methyl-protonated samples, this structure relied on a large number of difficult-to-assign side chain NOEs recorded on protonated samples to form inter-helix connectivities that resulted in extremely well-defined backbone and side chain conformations. Obtaining these crucial side chain assignments relied on the initial transfer of methyl information obtained on ILV methyl-protonated samples onto the other positions within those residues, followed by a gradual propagation of assignments onto further amino acids using protonated samples. This process was extremely laborious but resulted in a high quality structure determination of pSRII. In contrast, amide and methyl NOEs from an ILV labelled sample were not even sufficient to provide an adequate representation of the backbone fold and at best resulted in a loosely packed arrangement of the seven transmembrane helices with inter-helix distances 3–4 Å bigger than the values typically observed in structures of similar proteins deposited in the PDB (Bowie 1997).

Alternatives to NOE-based structural restraints are therefore desirable. Recently, lanthanide-induced pseudocontact shifts (PCSs) have been established as a structural tool for globular proteins (Liu et al. 2014a). Using pSRII as a model system (rotational correlation time for the protein-detergent complex of 34 ns at 308 K) we demonstrate here that a PCS-based approach using a lanthanide tag can be successfully applied for the structure determination of large integral membrane proteins.

Site-specific incorporation of paramagnetic lanthanide centres into a protein of interest produces various effects that can be utilised to provide structural information (Otting 2010). Paramagnetic relaxation enhancements (PREs) due to dipolar interactions with unpaired electrons provide long-range distance information. In practice, however, non-metal electron radical ligand tags are preferred, as used for example to provide some of the inter-helix distances in the structure determination of another 7TM protein, proteorhodopsin (Reckel et al. 2011). Partial alignment due to the anisotropic magnetic susceptibility of the metal moiety allows the measurement of residual dipolar couplings (RDCs), offering orientational information (Tolman et al. 1995). Pseudocontact shifts, as well as cross-correlated relaxation effects, provide both distance and orientational information as has been demonstrated on several globular proteins, mostly those containing a native metal binding site (Boisbouvier et al. 1999). PCSs are particularly attractive for use as structural restraints owing to this ability to give both long-range distance and orientational information, as well as the ease with which they can be recorded using only small quantities of protein and simple two-dimensional experiments.

A PCS represents the change in chemical shift of a nuclear spin resulting from its through-space dipolar interaction with the unpaired electron of the paramagnetic centre. The PCS depends on the distance and orientation of the nuclear spin with respect to the unpaired electron, as well as the magnetic susceptibility anisotropy of the paramagnetic centre, and is described by Eq. 1:

1 $$ {\Delta\delta }^{PCS} = \frac{1}{{12\pi r^{3} }}\left[ {\Delta \chi_{ax} \left( {3\cos^{2} \theta - 1} \right) + \frac{3}{2}\Delta \chi_{rh} \sin^{2} \theta  \cos 2\varphi } \right] $$

where Δχ_ax_ and Δχ_rh_ are the axial and rhombic components, respectively, of the anisotropic magnetic susceptibility tensor (Δχ tensor), *r* is the distance of the nucleus to the paramagnetic centre, and *θ* and *φ* are angles describing the orientation of the electron-nucleus vector with respect to the Δχ tensor frame. Therefore, if the coordinates defining the position of the paramagnetic centre, the Euler angles that relate the Δχ tensor frame to the molecular frame, and Δχ_ax_ and Δχ_rh_ are known, the PCS of a nuclear spin provides information on the location of the nucleus.

**Fig. 1:**
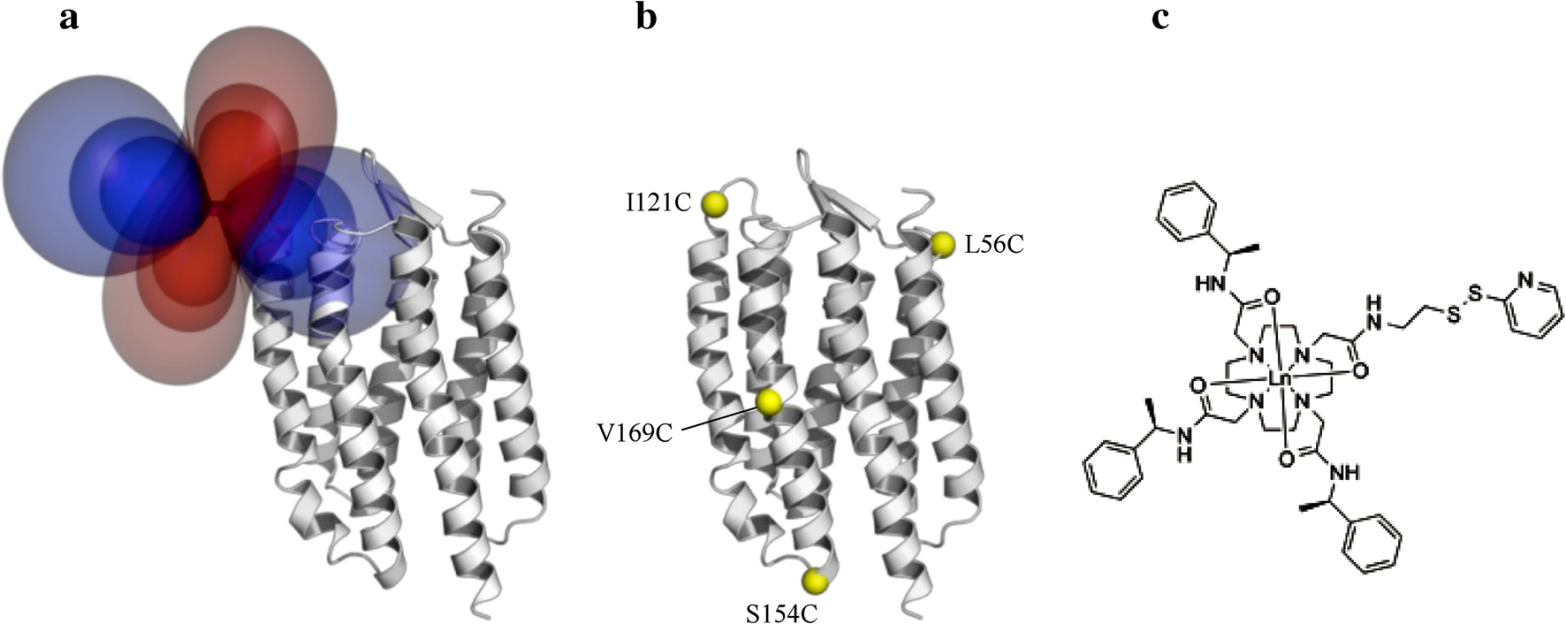
**a** Isosurfaces representing a hypothetical Δχ tensor for a paramagnetic lanthanide ion attached at the I121C position of pSRII, indicating spatial locations with identical PCS.* Red* and* blue lobes* indicate positive and negative contributions, respectively. The figure was prepared using the program Numbat (Schmitz et al. 2008). **b** The positions of the four single-cysteine mutations in pSRII introduced for lanthanide tag attachment via the cysteine side chain that were used to measure PCSs. **c** Structure of the C2 DOTA amide-based tag used to complex Dy^3+^, Tb^3+^, Tm^3+^, Yb^3+^ or Y^3+^

It is useful to visualise the Δχ tensor as isosurfaces of equal PCS values (Fig. 1a), where each isosurface shows the locations of a nuclear spin for which Eq. 1 predicts the same PCS value. Using this representation it is easy to see that to unambiguously determine the position of a nuclear spin, PCSs must be measured for different locations of the paramagnetic centre: with PCSs measured from just one position the nucleus could lie anywhere on the respective isosurface, while with PCSs measured from two positions the location of the nucleus is confined to the coordinates at which the two isosurfaces intersect. An increasing number of sites for the paramagnetic centre will further constrain the location of the nucleus, reducing ambiguity. It is therefore highly advantageous to use a labelling approach, which allows flexible positioning of the paramagnetic centre at different sites around the protein.

Several studies have made use of natural metal binding sites in proteins to incorporate lanthanide ions for PCS measurement (Allegrozzi et al. 2000; Pintacuda et al. 2004, 2006; Schmitz et al. 2006; Schmitz and Bonvin 2011; John et al. 2007). Alternatively a plethora of lanthanide-chelating peptides and small molecule tags have been developed which, in combination with genetic engineering, allow a more universal and flexible method for site-specific lanthanide incorporation (Liu et al. 2014a). Conformational mobility of the ligand sphere as well as motions of the entire metal complex relative to the protein will reduce the absolute values of the protein-derived tensor components and compromise the structural information that can be derived from the measured PCS values. Various strategies have been implemented with a view of reducing tag motion through attachment of a tag to two sites on the protein (Prudêncio et al. 2004; Vlasie et al. 2007; Keizers et al. 2007; Liu et al. 2012, 2014b), making use of a protein residue as an additional coordination group (Su et al. 2008; Man et al. 2010; Jia et al. 2011; Swarbrick et al. 2011a), using two tags to coordinate the metal (Swarbrick et al. 2011b), creating steric constraints by using a large tag (Graham et al. 2011; Loh et al. 2013) or by devising a more rigid ligand sphere (Häussinger et al. 2009).

PCSs induced by paramagnetic lanthanide ions have been used in the study of protein–protein (Pintacuda et al. 2006; Saio et al. 2010; Schmitz and Bonvin 2011; Hass and Ubbink 2014) and protein–ligand interactions (John et al. 2006; Zhuang et al. 2008; Saio et al. 2011; Guan et al. 2013), as well as in protein structure refinement (Allegrozzi et al. 2000), validation (de la Cruz et al. 2011, 2014; Chen et al. 2014) and determination when combined with Rosetta-based methods (Schmitz et al. 2012; Yagi et al. 2013; Shishmarev et al. 2013). However, to our knowledge, there are no current examples of using PCSs for the determination of membrane protein structures. Here we demonstrate the use of lanthanide tag-induced PCSs as a powerful tool for the global fold determination of pSRII, a seven-helical transmembrane sensor. The approach makes use of PCS restraints derived from a single cysteine side chain-attached, DOTA amide-based lanthanide-chelating tag (“C2”) positioned in four different locations on the protein, in combination with a limited set of NOEs from a methyl-protonated sample that alone is insufficient to correctly pack the helical bundle. Our results show that the overall fold of the backbone can be determined with an RMSD to the published pSRII NMR structure of 2.6 Å. While some variation is observed, depending on the set of NOEs used, the characteristic global fold of the 7TM protein is clearly established in all cases. Comparison to structures calculated solely with the same limited sets of NOEs (i.e., excluding any paramagnetic restraints) reveal that the PCS restraints drive the correct assembly of the helical bundle. Our study establishes the use of PCSs as a suitable tool to enable the backbone structure determination of membrane proteins under conditions where NOE data is sparse. While the strategy is demonstrated on the challenging case of a 7TM protein, the approach should be generally suited for membrane proteins and, in most cases, may still work under conditions where less NOE data is available than utilised here.

## Results

### Lanthanide tagging of pSRII

To investigate the suitability of lanthanide tag-induced pseudocontact shifts for the structure determination of membrane proteins, four pSRII mutants were prepared in which single cysteine residues were introduced at different positions around the protein (Fig. 1b). To assess any influence due to effects related to the local environment, mutation sites were chosen such that the lanthanide tags would reside in positions differing substantially in hydrophobicity; with L56C and I121C residing in extracellular loops, S154C in the cytoplasmic region and V169C in the transmembrane region of the protein. As wild-type pSRII does not contain any cysteines, no prior deletion mutagenesis was required. The single-cysteine mutants were attached via thio-disulphide exchange to C2, a DOTA amide-based lanthanide-chelating tag (Fig. 1c; de la Cruz et al. 2011), preloaded with one of the four paramagnetic lanthanide ions, Dy^3+^, Tb^3+^, Tm^3+^ or Yb^3+^, or the diamagnetic Y^3+^ as a reference. In the diamagnetic reference, only residues in the immediate vicinity of the Y^3+^-tag showed significant ^1^H and ^15^N chemical shift changes when compared to the WT form. Together with UV/vis spectra displaying the unchanged fine structure typical for the pSRII retinal absorption, this confirmed the structural integrity of the C2-tagged mutants.

### PCS measurement

**Fig. 2:**
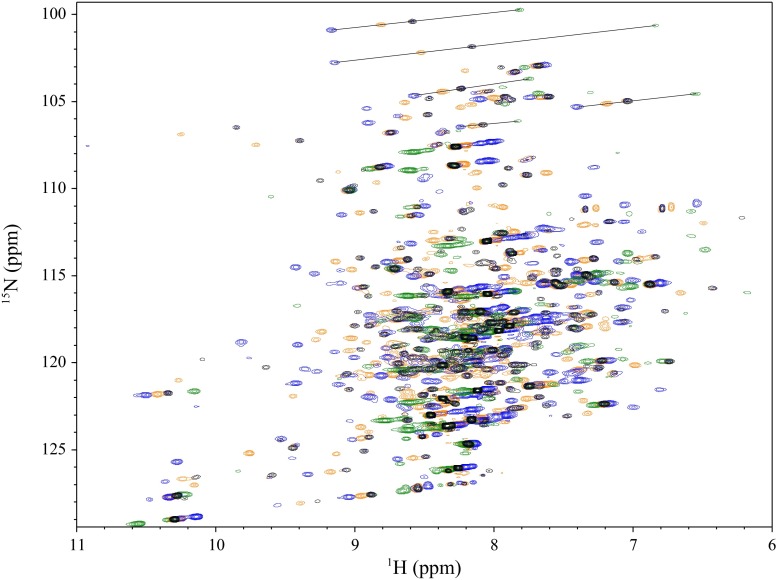
Superposition of 2D [^1^H,^15^N]-TROSY spectra recorded on C2-lanthanide-tagged pSRII V169C, for the diamagnetic reference Y^3+^ (*black*) and the paramagnetic metals Dy^3+^ (*green*), Tm^3+^ (*blue*) and Yb^3+^ (*orange*).* Lines* indicate a selection of observed PCSs. Spectra were recorded at 800 MHz ^1^H frequency and 308 K

2D [^1^H,^15^N]-TROSY spectra of the four single-cysteine pSRII mutants tagged with paramagnetic lanthanides demonstrated significant PCSs in all cases (Fig. 2 and Supplementary Fig. 1), with absolute values of up to 1.4 ppm measured relative to the diamagnetic references. The cross-peak assignments were transferred from wild-type pSRII spectra. This was assisted by the fact that PCSs in ^1^H and ^15^N dimensions are very similar in size, as the two nuclei are very close compared to the distance over which the dipolar interaction with the unpaired electron is active, resulting in peak displacements along nearly parallel lines. The use of multiple paramagnetic lanthanide ions can further facilitate assignment when the axial components of the Δχ tensors point in similar directions, and each metal can cause shifts to different extents and in different directions along these parallel lines, with Dy^3+^ and Tb^3+^ generally causing shifts in the opposite direction to Tm^3+^ and Yb^3+^. Together this allowed assignment of shifted cross-peaks even in relatively crowded spectral regions. From the four mutants using the four lanthanide ions a total of 737 PCSs were unambiguously assigned for backbone amides and the tryptophan side chain indoles, with at least one PCS assigned for 66 % of the residues. The numbers of assigned residues showing PCSs for each of the four mutants are given in Table 1. The residues affected by strong PRE broadening were all in the immediate vicinity of the lanthanide binding site but the extent of spectral bleaching varied depending on the lanthanide ion used, as described previously (Otting 2008). Similarly, owing to the different tensor sizes of the four lanthanide ions, the range over which measurable PCSs could be observed varied in each case, making the use of several paramagnetic ion tags complementary. Thus, using a range of different lanthanide ions in each position proved to be a necessity to improve the PCS-coverage of the protein, as well as providing the significant aforementioned advantage in facilitating residue assignments.

**Table 1:** Number of residues for which PCSs were measured on the four single-cysteine lanthanide tag-attached mutants of pSRII

Metal ion	L56C	I121C	S154C	V169C
Dy^3+^	57	51	45	26
Tb^3+^	58	46	42	–^a^
Tm^3+^	84	53	36	49
Yb^3+^	62	54	16	58

^a^pSRII-V169C was not tagged with C2–Tb^3+^

### NOE measurement

In contrast to our previous comprehensive structure determination (Gautier et al. 2010), only a small subset of NOEs was used from 3D ^13^C- and ^15^N-separated NOESY experiments, recorded on a highly-deuterated, uniformly ^15^N and selectively ^13^CH_3_-labelled ILVA (Godoy-Ruiz et al. 2010) sample of pSRII (with LV ^13^CH_3_/^12^CD_3_ labelled). Amide-amide, methyl–methyl and methyl-amide NOEs were assigned, resulting in a total of 531 unambiguous NOEs. To assess the impact of the PCS restraints on structure calculations this initial data was used to prepare limited NOE sets. The original NOE list was filtered according to the individual residue types of the ILVA sample, so that reduced sets contained only NOEs involving the methyl groups of one particular amino acid type, and all of the inter-amide NOEs (Table 2). Filtering using the residue type as a criterion only served the purpose of reducing the number of NOEs in the calculations in order to assess the impact of PCS restraints. Equally for this purpose, subsets with a limited number of randomly selected NOEs could have been generated to imitate a sparse NOE situation. Depending on the amino acid type, the generated distance restraint lists varied between 221 and 282 NOEs, while the number of inter-helix NOEs varied between 4 and 19.

**Table 2:** Unambiguous NOEs from 3D ^13^C- and ^15^N-separated NOESY experiments measured on a highly-deuterated, uniformly ^15^N-, and selectively ^13^CH_3_-labelled ILVA sample of pSRII

NOE list	NH–NH	CH_3_–NH	CH_3_–CH_3_	Total	Inter-helix
All ILVA	180	282	69	531	91
Isoleucine	180	39	2	221	4
Leucine	180	60	7	247	19
Valine	180	98	4	282	8
Alanine	180	85	6	271	10

Data were recorded at 800 MHz ^1^H frequency and 308 K

### Dihedral angles and hydrogen bonds

Additional restraints in the form of chemical shift-derived backbone dihedral angles and hydrogen bonds were included to support helix formation. Dihedral angles were predicted from ^15^N, ^13^C_α_, ^13^C_β_ and ^13^C’ chemical shifts using the program TALOS (Cornilescu et al. 1999), giving 190 φ and ψ angles. Hydrogen bonds were predicted based on secondary structure analysis from chemical shift information and solvent accessibility experiments, which identified solvent exchange-protected amide protons. This gave 132 predicted hydrogen bonds exclusively within the transmembrane helices and in the β-sheet region of the B–C loop. Structures calculated using only dihedral angles and hydrogen bonds revealed the correct formation of the individual secondary structure elements but no recognisable packing of the helices.

### Structure calculations using limited sets of NOEs

**Fig. 3:**
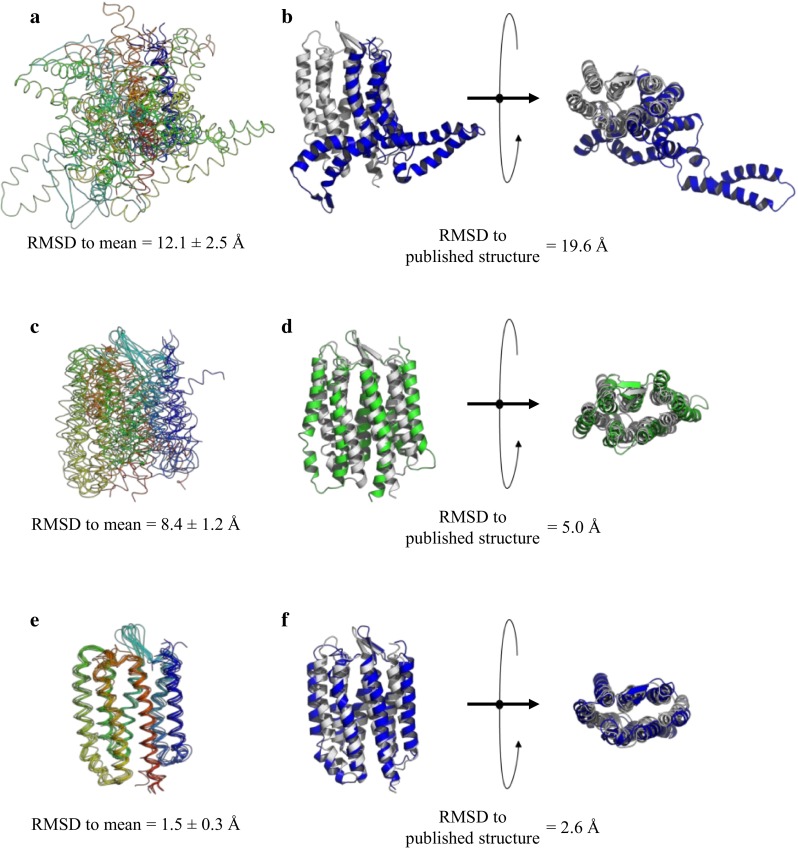
Results of pSRII backbone structure calculations using different sets of restraint types. **a** Superposition of the eight lowest energy structures (20 %) based on the leucine-filtered limited NOE set (see Table 2). No PCS restraints were included in the calculations. **b** Superposition of the structure closest to the mean from **a** in *blue* and the equivalent structure from the published high resolution NMR structure ensemble (Gautier et al. 2010) in *grey*. **c** Superposition of the eight lowest energy structures (20 %) calculated using 737 PCSs (see Table 1), in the absence of any NOE restraints. **d** Superposition of the structure closest to the mean from **c** in *green* and the equivalent structure from the published high resolution NMR structure ensemble in grey. **e** Superposition of the eight lowest energy structures (20 %) calculated with the limited leucine-filtered NOE set used in **a** and the full PCS set used in **c**. **f** Superposition of the structure closest to the mean from **e** in *blue* and the equivalent structure from the published high resolution NMR structure ensemble in *grey*

Structures were first calculated without the inclusion of PCS restraints. Limited NOE sets containing inter-amide NOEs and additional contacts to methyl groups of only one residue type were used in combination with 190 φ and ψ angles and 132 hydrogen bond restraints. The calculations resulted in poor quality structures in terms of both precision and accuracy, regardless of the NOE set used. Even in the best case, using an NOE set based on leucine methyl groups containing 247 NOEs with 19 inter-helix restraints, the ensemble RMSD for the eight (20 %) lowest energy structures from these calculations was high at 12.1 ± 2.5 Å, demonstrating poor precision (Fig. 3a). Comparing the structure closest to the mean of this ensemble with the analogous structure in the published ensemble, the RMSD was also high at 19.6 Å, indicating failure to define a 7TM fold based on these restraints (Fig. 3b). The calculations produced similar or even worse results using limited NOE sets involving other residue types (Supplementary Fig. 2).

### Structure calculations using PCS

To assess the power of the measured PCS restraints, structures were calculated in the complete absence of NOE information. Thus, the restraints used were 190 φ and ψ angles, 132 hydrogen bonds, and 737 PCSs from the four lanthanide-tagged mutants. The calculations were otherwise identical in input to those presented above. Calculated structures were not of high quality, but were significantly better than those obtained using only the limited NOE sets, with a recognisable 7TM fold (Fig. 3c) and an RMSD of 5.0 Å to the published structure for the structure closest to the mean (Fig. 3d).

### Structure calculations using PCS and limited sets of NOEs

Finally, to assess the quality of structures in a realistic scenario in which both PCS restraints and a limited number of NOEs are available, structures were calculated using each reduced NOE set in combination with the full set of PCSs. Thus, the restraints used were 190 φ and ψ angles, 132 hydrogen bonds, 737 PCSs from the four lanthanide-tagged mutants and NOEs based on methyl groups of a particular residue type. Again, other than the number of restraints used, the calculations were identical in input to those presented above. In the best case, using the NOE set based on leucine methyl groups, the eight (20 %) lowest energy structures overlaid well, with an ensemble RMSD of 1.5 ± 0.3 Å (Fig. 3e), and the closest structure to the mean overlaid relatively well with the published NMR structure (Gautier et al. 2010), with an RMSD of 2.6 Å (Fig. 3f). These improvements in precision and accuracy relative to the structures calculated without PCSs are significant and demonstrate the power of these restraints for packing the preformed helices into the bundle. Using PCSs in combination with other limited NOE sets also improved structures dramatically compared to those derived from these NOEs alone (Supplementary Fig. 3). In order to assess if there was redundancy in the PCS data, similar calculations were repeated using either only PCS information measured with the weaker shifting Yb^3+^ or stronger shifting Dy^3+^ loaded tag, respectively. However, in both cases these reduced sets of PCS restraints did not succeed in driving the structure calculations towards an accurate fold.

## Discussion

Structural studies of membrane proteins are challenging, and overcoming a shortage of NOEs due to the high levels of deuteration needed to study sizeable membrane proteins solubilized in a membrane-mimicking environment can be a serious obstacle in the global fold determination. Even in the presence of selectively protonated samples including e.g. ILV methyl-protonation, there are often insufficient inter-helix NOE contacts to obtain the global fold of a polytopical α-helical membrane protein. This situation can be improved by the inclusion of alternative types of structural restraints. In this contribution we investigated the suitability of PCSs as an alternative or complementary type of restraint for membrane protein structure determination, using sensory rhodopsin II solubilised in c7-DHPC micelles as a model case. Our results demonstrate clearly the power of lanthanide tag-derived PCSs as structural restraints in driving the formation of the pSRII tertiary structure. The PCS restraints successfully pack the bundle of helices, which individually are stabilised by dihedral angles, hydrogen bonds and a subset of short-range NOEs.

We used the 7TM helical pSRII sensory receptor as a model case since the results of this study can be directly compared against the recent NOE-based full NMR structure determination performed in our laboratory (Gautier et al. 2010), but also because obtaining a global fold of a polytopical α-helical MP represents a particularly demanding case. Our previous work showed that a large number of NOEs derived from a multitude of samples, including protonated ones, resulted in a high-quality structure of pSRII. Obtaining such a large number of NOEs was very laborious and assigning thousands of NOEs was very challenging. While these efforts were aimed at obtaining best-defined backbone and side chain conformations, earlier attempts using NOEs from an ILV methyl-protonated sample were not even sufficient to provide an adequate representation of the backbone fold, revealing an insufficiently defined arrangement of the 7TM helices, with inter-helix distances 3–4 Å larger than found for the deposited NMR structures. Based on this scenario, we concluded that a potential typical situation might be one where, for a given protein study, only a limited number of NOEs might be available, as likely provided by a highly-deuterated ILV or ILVA methyl-protonated sample. As these restraints may be insufficient to define a meaningful tertiary fold, they would have to be supplemented by alternative restraints. Thus, the use of PCSs as additional restraints, as demonstrated here for pSRII (*τ*_*c*_ ~34 ns), is likely to be well-suited for other large membrane proteins.

Using various combinations of restraint types, different rounds of structure calculations were run in order to assess the impact of the PCSs. The results of the structure calculations based only on limited NOE sets containing inter-amide NOEs and NOEs to methyl groups of only one residue type bore very little resemblance to the deposited NMR structure (Fig. 3a, b and Supplementary Fig. 2). In contrast, calculating structures using the available PCS data in the complete absence of NOE restraints resulted in an already recognizable 7TM fold, with a closest-to-the-mean structure RMSD of 5.0 Å to the published NMR structure (Fig. 3c, d). Finally, using a combination of the limited NOE sets that are inadequate to define the 7TM architecture of pSRII and all the PCS restraints, the structures are significantly improved and overlay relatively well with the published structure (Fig. 3e, f and Supplementary Fig. 3).

The major contribution of the PCSs is to pack the bundle of helices, which individually are stabilized by dihedral angles and hydrogen bonds. Long-range NOEs also play a role in the packing of the helices, and hence the combination of the PCSs with the limited sets of NOEs improved the results of the previous calculations. A larger number of inter-helix NOEs is beneficial, meaning the leucine set including 19 inter-helix NOEs led to the best global fold, showing an RMSD of 2.6 Å to the published NMR structure (Fig. 3e, f).

The calculations used PCSs measured from four different positions distributed over the protein. Using PCSs from multiple positions is important for pinpointing the exact location of a nucleus, and also in achieving good PCS coverage throughout the protein structure. Structures of pSRII calculated with PCSs from fewer positions were significantly poorer, highlighting the importance of using tags at multiple positions. A more careful positioning of the lanthanide tags through a choice of different mutants, increasing their number or possibly including more transmembrane locations instead of loop region mutants (vide infra) might improve these results further.

At the same time our calculations showed also that the use of several lanthanide ions was required to increase the number of residues affected by PCSs and to improve the number of assignable peaks in the spectra. Similar calculations relying only on PCS data from the Yb^3+^ or Dy^3+^ probes and including the information from the limited NOE sets resulted in poor quality structures. Analysis of the distances over which the PCSs are observed (Supplementary Fig. 4) confirmed that the tags of the stronger shifting lanthanide ion Dy^3+^ are contributing a substantial number of longer distance restraints that are beyond the reach of the Yb^3+^ probes, while at the lower distance end due to reduced PRE bleaching the Yb^3+^ tags provide a sizeable amount of shorter distance restraints that are not obtainable with the stronger shifting lanthanide ions. In order to achieve as complete a PCS coverage as possible, to assist with peak assignments and to reduce data loss from coincidental peak overlap, it is therefore recommended to use multiple lanthanide ion probes. In our case the data based on four lanthanide ion probes proved to be adequate.

Observed PCSs for the transmembrane mutant V169C were generally larger than for the three loop region mutants. Most likely this results from differences in the dynamics, as motion of the tag reduces the effective Δχ tensor components. Thus, the tags attached in the transmembrane region at V169C appear to show reduced dynamics, either through steric constraints with the detergent micelle or favourable hydrophobic interactions between the ligand side chains and the protein surface. Rigid tag attachment is advantageous for recording PCSs since their distance dependence means that, with significant motion, a single averaged Δχ tensor may not fit PCSs at different distances from the tag (Shishmarev and Otting 2013). Hence, tag attachment in transmembrane regions may provide a method to reduce tag motion and so improve the value of PCSs measured from single cysteine-attached probes.

Δχ_ax_ and Δχ_rh_ values are required for structure calculations using PCSs (Eq. 1). These are commonly determined by fitting experimental PCSs to a known protein structure. However, for *de novo* structure determination, as was simulated here, such an approach is clearly not possible and Δχ_ax_ and Δχ_rh_ values must be obtained in a different way. In principle they can be fitted iteratively during the structure calculations (Banci et al. 2004), however this was unsuccessful here as no convergence of the tensor values was observed (data not shown). Instead, all structure calculations relied on literature known values for Δχ_ax_ and Δχ_rh_ obtained for the same lanthanide ions attached via a “C1” tag (the enantiomer of the C2 tag used here) to the *E. coli* arginine repressor (Graham et al. 2011). With the tensor values being largely determined by the nature of the lanthanide, and in view of the similarity of the C1 and C2 ligand spheres, these values were considered as acceptable approximations. However, motion of the tag will also affect the effective Δχ_ax_ and Δχ_rh_ values, and may vary for attachment to different proteins and for different sites within a protein, as discussed above. Nevertheless, in our hands, using published Δχ_ax_ and Δχ_rh_ values for all tagged mutants enabled the PCSs to drive the calculations towards the correct global fold of pSRII, with the calculated structures satisfying the PCS restraints well (Supplementary Fig. 5) and motional averaging of the tensor components not apparently reflected in increased PCS violations. The stabilisation of individual helices by other restraints, as well as the small number of inter-helix NOEs, may compensate for inaccuracies in the Δχ_ax_ and Δχ_rh_ values.

As a proof of the PCS data quality, Δχ_ax_ and Δχ_rh_ values were also determined by fitting the measured PCSs to the published pSRII NMR structure using the program Numbat (Schmitz et al. 2008; Supplementary Table 1). Δχ_ax_ and Δχ_rh_ values fitted in this way were similar to the literature values used above, although values for metals bound at V169C were generally larger in magnitude than those for other tag positions, again suggesting reduced mobility of the transmembrane-attached tags. Fitting gave good correlations between experimental and back-calculated PCSs in all cases (Supplementary Fig. 6). Structures calculated using the fitted Δχ_ax_ and Δχ_rh_ values were similar in quality to those presented above (data not shown) demonstrating an inherent robustness of the method against minor tensor changes.

## Conclusions

The results presented here demonstrate the power of lanthanide tag-derived PCSs as a tool for membrane protein structure determination. The PCSs are crucial in driving the global fold of the seven-helical membrane protein pSRII, a task which can be highly challenging using only NOEs due to a lack of sufficient contacts between adjacent helices. The ease with which PCSs can be measured and assigned using simple two-dimensional experiments recorded on ^15^N-labelled samples makes such an approach highly attractive for challenging systems and will be of great value in determining the backbone structure of large polytopical membrane proteins. There are indications that lanthanide tags attached to transmembrane regions of a protein are less affected by dynamics, improving the value of PCSs measured under such conditions.

## Methods

### C2-tagged pSRII sample preparation

The C2 tag was synthesised and loaded with lanthanide metal ions as described previously (Graham et al. 2011). Uniformly ^15^N-labelled single-cysteine pSRII mutants (L56C, I121C, S154C and V169C) were expressed in *E. coli* and purified following previously described protocols (Gautier et al. 2010) with 10 mM DTT included in purification buffers to maintain cysteines in a reduced state. For tagging reactions, DTT was removed using a PD10 column (GE Healthcare) and the pSRII sample was concentrated to a concentration of 300–400 μM using a Vivaspin centrifugal concentrator with a molecular weight cut-off of 10 kDa (Sartorius). The pSRII solution in tagging buffer (20 mM Na phosphate pH 7, 50 mM NaCl) was added to a sevenfold molar excess of C2 tag preloaded with the relevant lanthanide ion (Dy^3+^, Tb^3+^, Tm^3+^, Yb^3+^ or Y^3+^) in the same buffer, and the mixture was incubated at room temperature for 18 h. Unreacted free tag was removed using a PD10 column, and the sample was washed in NMR buffer (20 mM Na phosphate pH 6, 50 mM NaCl) containing 0.1 % C7-DHPC by repeated rounds of concentration and dilution. Final NMR samples were at a concentration of 300–400 μM in NMR buffer containing 3 % C7-DHPC.

### ILVA methyl-protonated pSRII sample preparation

*U*-[^2^H,^15^N] Ileδ1-[^13^CH_3_] Leu,Val-[^13^CH_3_,^12^CD_3_] Ala-[^13^CH_3_]-pSRII was expressed following adaptation of cells by subculturing at 37 °C in M9 minimal medium with increasing D_2_O content (50, 70, 90, 98 %). *U*-[^2^H,^13^C]-glucose and ^15^NH_4_Cl were used as carbon and nitrogen sources in final cultures and these cultures were supplemented with 2.5 g l^−1^ succinic acid-2,2,3,3-d_4_, 170 mg l^−1^ 2-keto-3-(methyl-d_3_)-butyric acid-4-^13^C,3-d_1_; 80 mg l^−1^ 2-ketobutyric acid-4-^13^C,3,3-d_2_ (all Sigma Aldrich) and 500 mg l^−1^ l-alanine 3-^13^C, 2-d (Cambridge Isotope Laboratories) 1 h prior to induction. Expression was induced at an OD_600_ of 1 by addition of 1 mM IPTG and 10 μM all-*trans* retinal and cultures were incubated at 30 °C for a further 8 h with supplementation every 2 h with 10 μM all-*trans* retinal. Purification followed the same protocol used above for C2-tagged pSRII and described previously (Gautier et al. 2010). The final NMR sample was at a concentration of 580 μM in NMR buffer containing 3 % C7-DHPC.

### NMR spectra

NMR spectra for measuring PCSs and NOEs were recorded at 35 °C and 800 MHz on Bruker DRX 800 and AVIII 800 spectrometers equipped with a 5 mm TXI HCN cryoprobe. For measuring PCSs 2D [^1^H,^15^N]-TROSY spectra were recorded on uniformly ^15^N-labelled single-cysteine pSRII mutants tagged with the C2-tag with bound paramagnetic lanthanide (Dy^3+^, Tb^3+^, Tm^3+^ and Yb^3+^) and diamagnetic Y^3+^. For measuring NOEs, a 3D ^13^C-separated [^1^H,^1^H]-NOESY HMQC spectrum and a 3D ^15^N-separated [^1^H,^1^H]-NOESY TROSY spectrum were recorded on ILVA methyl-protonated pSRII. Experiments for the prediction of dihedral angles and hydrogen bonds were recorded as described previously (Gautier et al. 2010).

### Structure calculations

Structure calculations were performed using simulated annealing methods implemented in the software Xplor-NIH (Schwieters et al. 2003) and using the PARArestraints set of modules for inclusion of PCS restraints (Banci et al. 2004). 190 backbone φ and ψ angles and 132 backbone hydrogen bond restraints were included in all calculations, with unambiguous amide–amide, amide-methyl and methyl–methyl NOEs based on individual ILVA residue types and 737 PCSs also included as indicated. NOEs were converted to distances using CcpNMR analysis and were given a tolerance of ±20 %. PCS tolerances were set to ±10 %. Hydrogen bonds were defined by artificial N–O and H–O NOEs with distances of 3.3 and 2.3 Å respectively. The retinal chromophore was omitted from all calculations since additional protein-retinal NOEs are required to maintain its correct position within the helix bundle.

Other than the number of restraints and specific details relating to the inclusion of different restraint types, the input for all calculations was identical. For inclusion of PCSs, Δχ tensors were defined by pseudoatoms, which were free to move during calculations to allow the position of the metal centre and orientation of the principle axis system to be fitted. The origin pseudoatom was held 12 ± 2.5 Å from the C_β_ of the tag attachment residue during the calculation by an artificial NOE. This was calculated as the correct approximate distance assuming the linker adopts an extended conformation. Δχ_ax_ and Δχ_rh_ values were taken from values previously published for the same lanthanide ions attached via the C1 tag (the enantiomer of the C2 tag used here) to the *E. coli* arginine repressor (ArgN) (Graham et al. 2011) and had values, in units of 10^−32^ m^3^, of Δχ_ax_: Dy^3+^ = −29, Tb^3+ ^= −27, Tm^3+^ = 37, Yb^3+^ = 13, Δχ_rh_: Dy^3+^ = −11, Tb^3+^ = −4, Tm^3+^ = 12, Yb^3+^ = 3.

All structures were calculated from a fully extended starting structure by an initial minimisation step in Cartesian space using only empirically-derived restraints, followed by simulated annealing steps in torsion angle space using all empirical and experimental restraints. Simulated annealing steps consisted of 200 ps dynamics at 10,000 K followed by slow cooling to 100 K in steps of 25 K. Forty structures were calculated in each case and an ensemble of the 20 % lowest energy structures was taken for analysis. The ensemble RMSD was calculated as the average backbone RMSD of each structure to the mean structure of the ensemble. The RMSD to the published NMR structure (PDB accession code 2KSY) was calculated for the closest structure to the mean of the ensemble in each case. All RMSDs were calculated for backbone atoms only and for residues 1–220 which excludes the flexible C-terminus.

## Electronic supplementary material

Below is the link to the electronic supplementary material.

Supplementary material 1 (PDF 3052 kb)
