# Supplementary material for: Integral membrane protein structure determination using pseudocontact shifts
Source: J Biomol NMR. 2015 Jan 22;61(3):197–207. doi: 10.1007/s10858-015-9899-6 (PMC4412549; doi:10.1007/s10858-015-9899-6)
Supplement: Supplementary file 1 — Supplementary material 1 (PDF 3052 kb) [file 10858_2015_9899_MOESM1_ESM.pdf]

Supplementary Information

Integral membrane protein structure determination using pseudocontact shifts

Duncan J. Crick[a], Jue X. Wang[a], Bim Graham[b], James D. Swarbrick[b], Helen R. Mott[a] and Daniel Nietlispach[a*]

[a] Department of Biochemistry, University of Cambridge, UK

[b]Monash Institute of Pharmaceutical Sciences, Monash University, Australia

***corresponding author:**

email: dn206@cam.ac.uk

phone: +44 1223 766023

fax: +44 1223 766002

Supplementary Figure 1

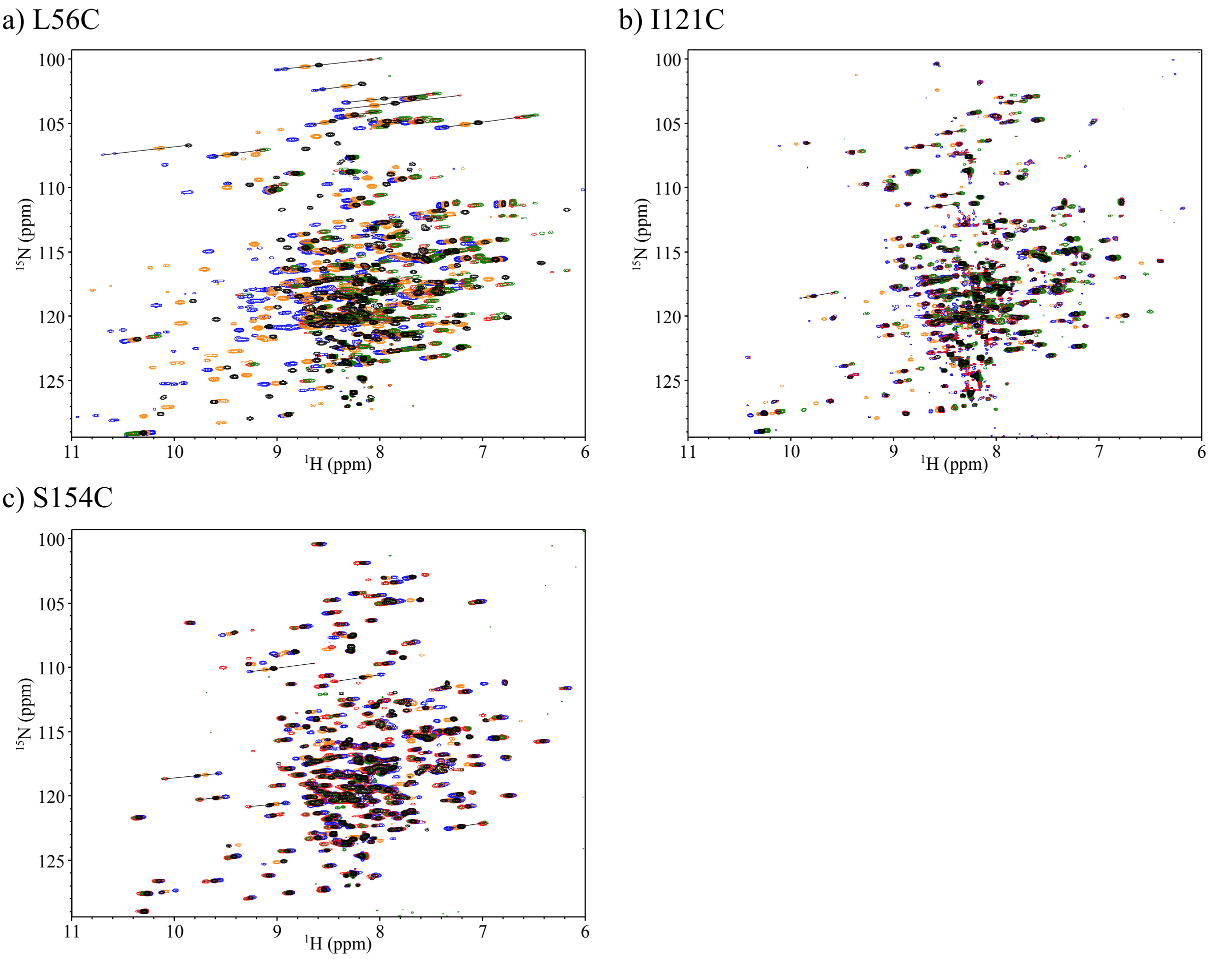

Supplementary Figure 1. Superposition of 2D [^{1}H,^{15}N]-TROSY spectra recorded on C2-lanthanide-tagged pSRII for the diamagnetic reference Y^{3+} (black) and the paramagnetic metals Dy^{3+} (green), Tb^{3+} (red), Tm^{3+} (blue) and Yb^{3+} (orange). Tags were attached at **(a)** L56C, **(b)** I121C and **(c)** S154C. Lines indicate a selection of observed PCSs. Spectra were recorded at 800 MHz ^{1}H frequency and 308 K.

Supplementary Figure 2

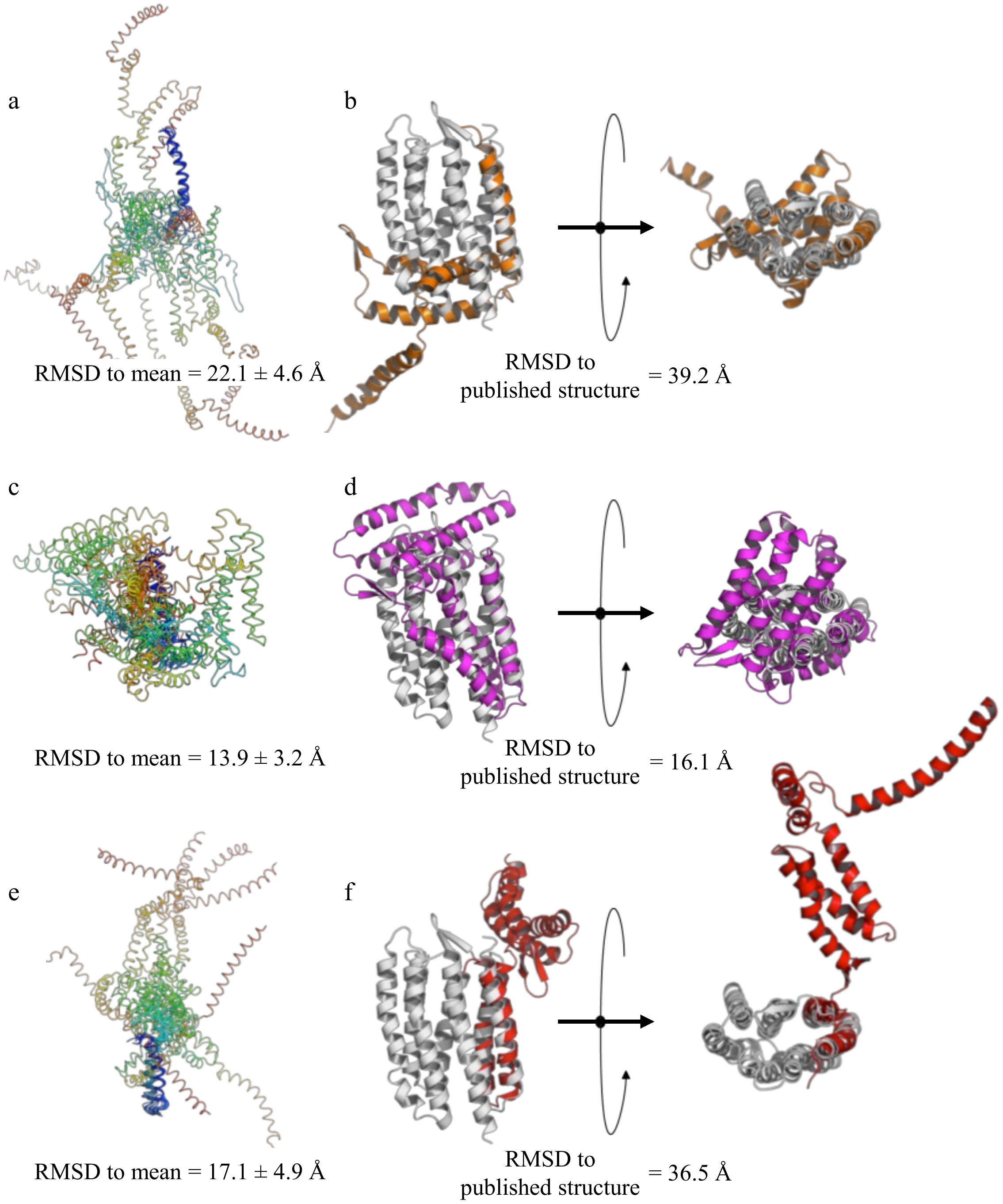

Supplementary Figure 2. Results of pSRII backbone structure calculations using limited sets of NOEs based on the methyl groups of isoleucine **(a-b)**, valine **(c-d)** and alanine **(e-f)** residues. No PCS restraints were included in the calculations. (a), (c) and (e) show superpositions of the eight lowest energy structures (20%), and (b), (d) and (f) show superpositions of the structure closest to the mean from the respective ensemble (coloured) and the equivalent structure from the published high resolution NMR structure ensemble (grey; Gautier *et al*, 2010).

Supplementary Figure 3

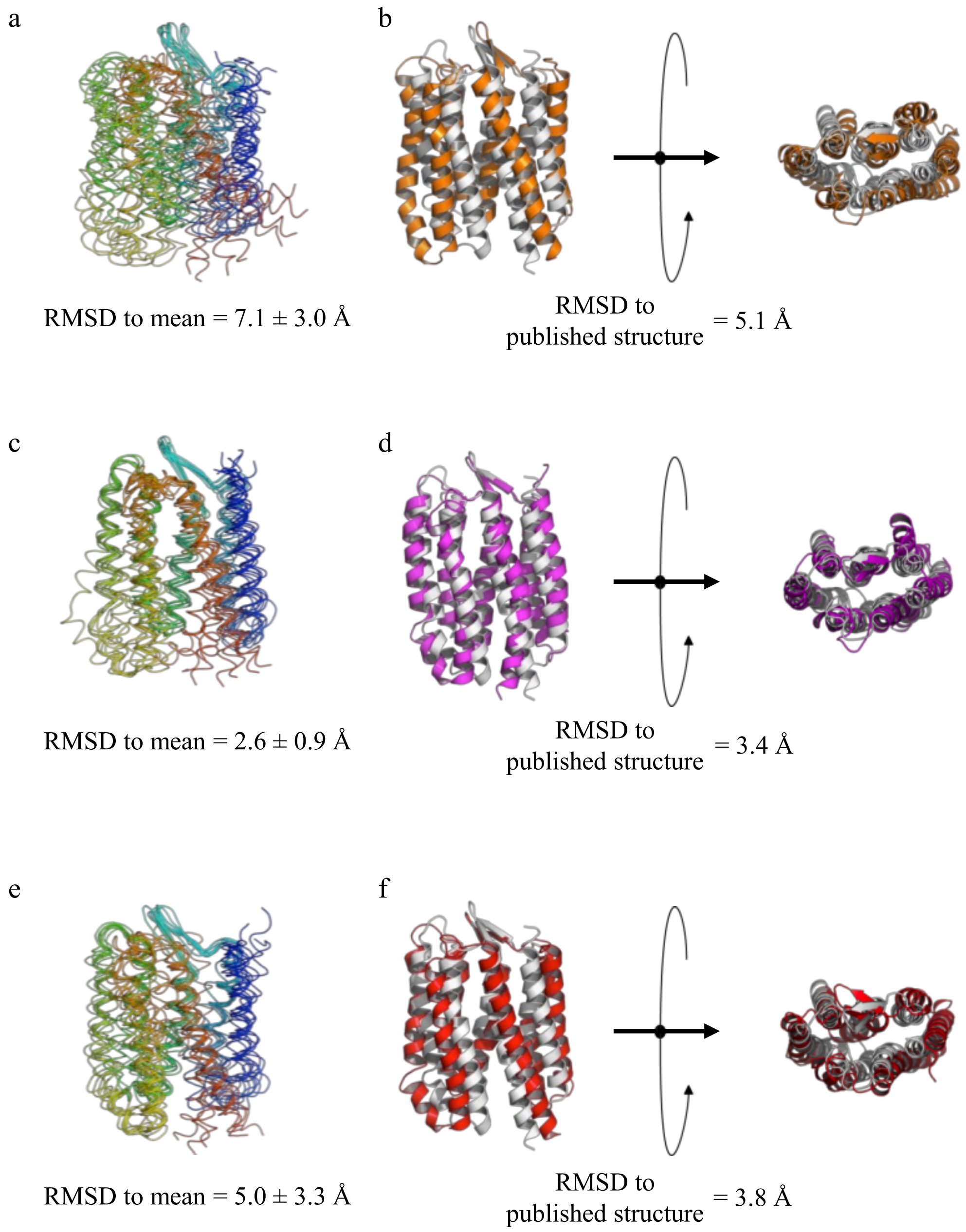

Supplementary Figure 3. Results of pSRII backbone structure calculations using PCS restraints in combination with limited sets of NOEs based on the methyl groups of isoleucine **(a-b)**, valine **(c-d)** and alanine **(e-f)** residues. (a), (c) and (e) show superpositions of the eight lowest energy structures (20%), and (b), (d) and (f) show superpositions of the structure closest to the mean from the respective ensemble (coloured) and the equivalent structure from the published high resolution NMR structure ensemble (grey; Gautier *et al*, 2010).

Supplementary Figure 4

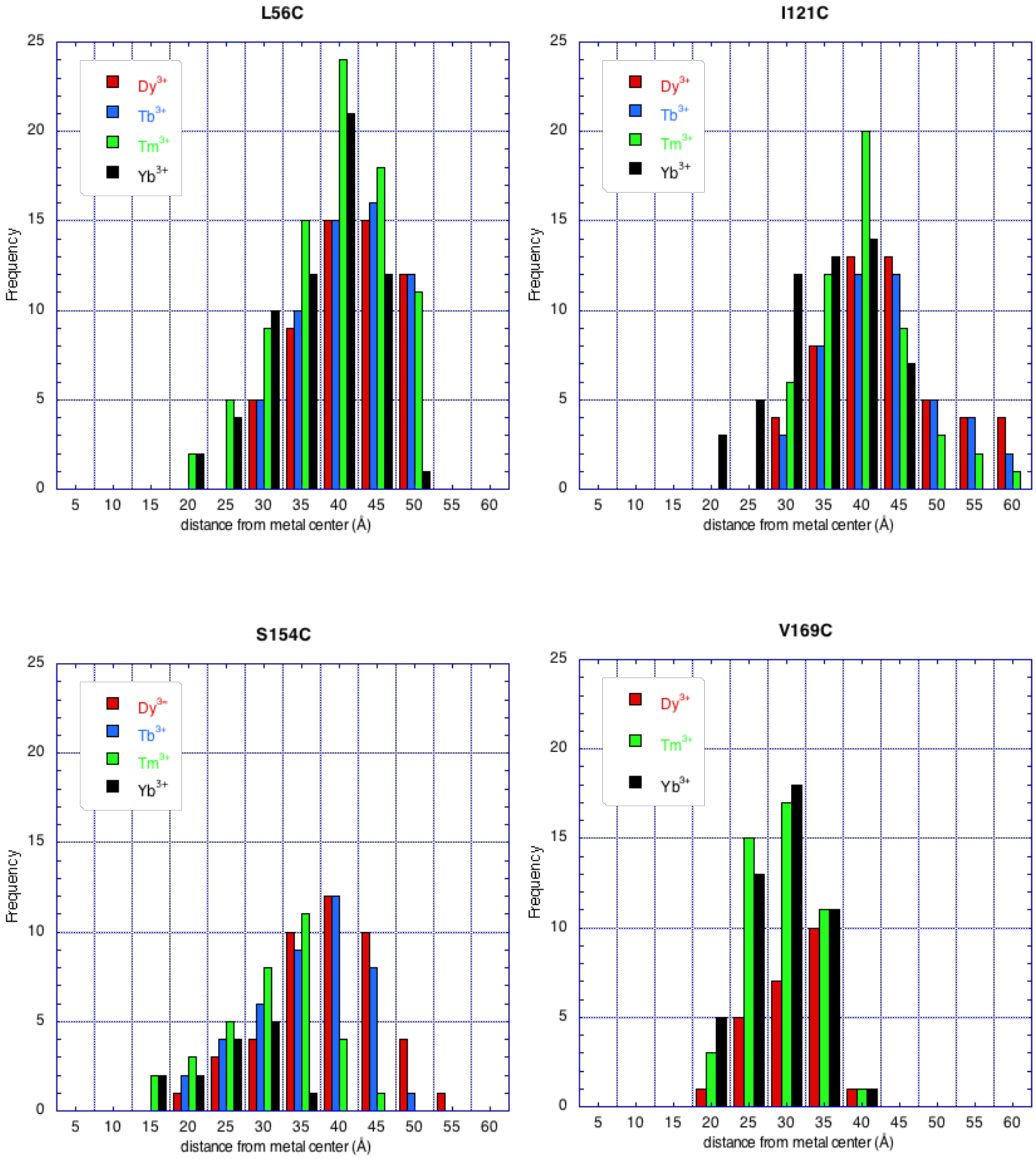

Supplementary Figure 4. Histograms showing the frequency of experimentally observed PCSs plotted against the amide proton to lanthanide ion distance over which they occur. The observed PCSs are grouped in 5 Å bins and are shown for the structured residues 1 – 222 for the mutants L56C, I121C, S154C and V169C with attached C2 tag loaded with the lanthanide ions Dy^{3+}, Tb^{3+}, Tm^{3+} and Yb^{3+}, respectively.

Supplementary Figure 5

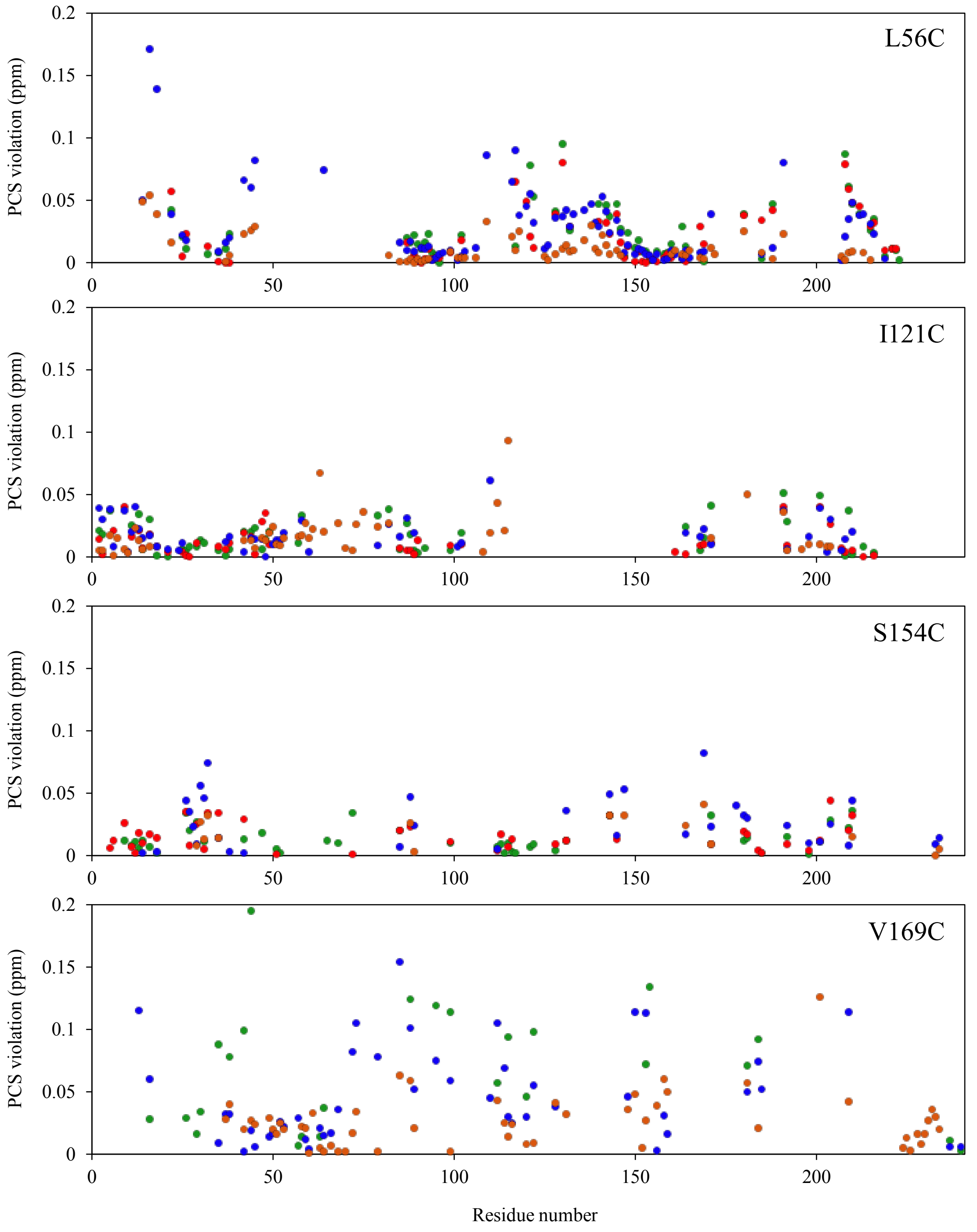

Supplementary Figure 5. Absolute sizes of violations for PCSs from Dy^{3+} (green), Tb^{3+} (red), Tm^{3+} (blue) and Yb^{3+} (orange) for the closest structure to the mean of the ensemble calculated using PCSs with a limited NOE set based on leucine methyl groups (see Figure 3f in the main text). Violations are calculated as the difference between experimental shifts and those predicted based on the calculated structure and the inputted tensor values.

Supplementary Figure 6

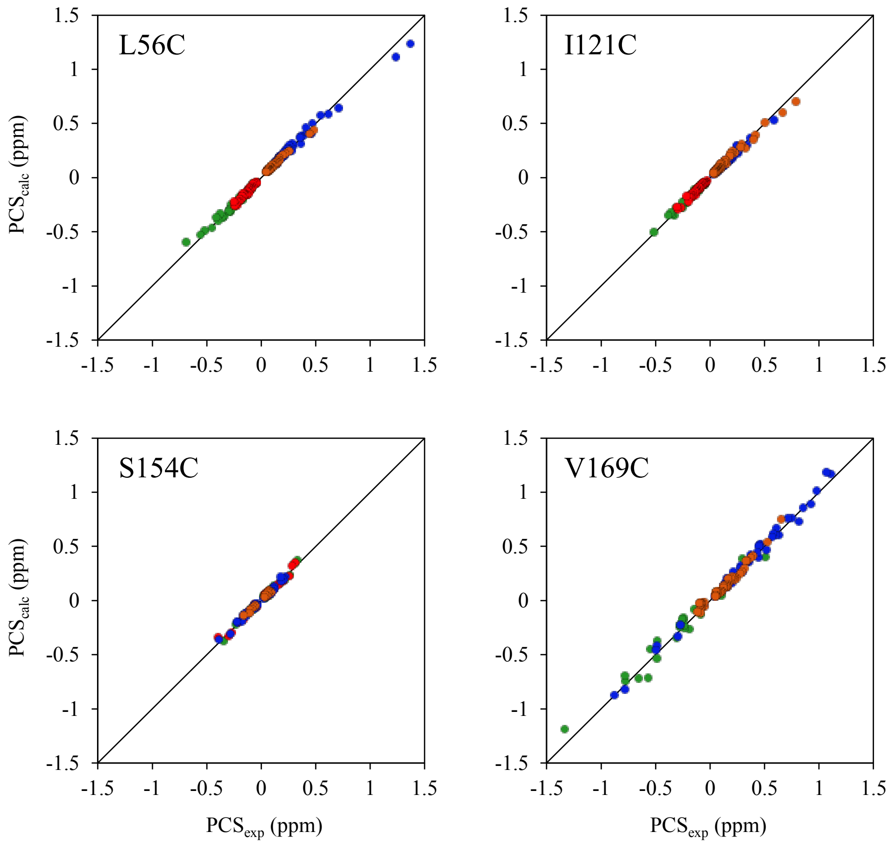

Supplementary Figure 6. Correlations between experimental PCSs and those back-calculated from $\Delta\chi$ tensors fitted to the pSRII NMR structure (see Supplementary Table 1) using the program Numbat (Schmitz *et al*, 2008) for PCSs from Dy^{3+} (green), Tb^{3+} (red), Tm^{3+} (blue) and Yb^{3+} (orange).

Supplementary Table 1. $\Delta\chi$ tensors obtained by fitting the experimental PCSs to the pSRII NMR structure using the program Numbat (Schmitz *et al*, 2008). PCSs for all metals at a particular mutant position were fitted simultaneously thus giving a common metal position. Errors represent one standard deviation from mean tensor values obtained using a Monte Carlo analysis of 100 trials that randomly omitted 20% of the PCS data. Q-factors were calculated as the RMSD between measured and predicted PCSs divided by the RMS of the measured PCSs. The orientations of the tensor axes are shown as coordinates representing unit vectors with respect to the origin.

pSRII mutant (tag attachment site)	Metal ion	$\Delta\chi_{ax}$ (10^{-32} m^3)	$\Delta\chi_{rh}$ (10^{-32} m^3)	Q	Coordinates of tensor axes	Coordinates of metal position
L56C	Dy^{3+}	-36 ± 1	-4 ± 2	0.09	x -0.470 0.775 0.422<br>y -0.293 -0.588 0.754<br>z 0.833 0.231 0.503	21.316 -18.420 -10.691
	Tb^{3+}	-31 ± 1	-5 ± 2	0.09	x -0.472 0.753 0.458<br>y -0.221 -0.604 0.765<br>z 0.853 0.260 0.452	
	Tm^{3+}	25 ± 1	6 ± 1	0.09	x 0.649 0.660 -0.379<br>y -0.466 0.738 0.487<br>z 0.601 -0.139 0.787	
	Yb^{3+}	9 ± 1	2 ± 1	0.08	x 0.683 0.660 -0.314<br>y -0.482 0.730 0.485<br>z 0.549 -0.180 0.816	
I121C	Dy^{3+}	-25 ± 8	-10 ± 4	0.08	x 0.492 0.720 0.489<br>y -0.827 0.563 0.004<br>z -0.272 -0.406 0.872	29.065 19.569 4.640
	Tb^{3+}	-19 ± 8	-6 ± 4	0.09	x -0.358 -0.284 0.889<br>y 0.489 0.754 0.438<br>z -0.795 0.592 -0.131	
	Tm^{3+}	18 ± 2	3 ± 3	0.10	x 0.515 0.704 0.490<br>y -0.179 0.647 -0.741<br>z -0.838 0.294 0.459	
	Yb^{3+}	9 ± 8	4 ± 5	0.11	x 0.287 0.555 -0.781<br>y 0.443 0.646 0.622<br>z 0.849 -0.525 -0.060	

(Supplementary Table 1 continued)

S154C	Dy^{3+}	-21 ± 1	-7 ± 1	0.12	x 0.583 -0.806 0.102<br>y 0.704 0.439 -0.559<br>z 0.406 0.397 0.823	-14.264 5.773 18.978
	Tb^{3+}	-18 ± 1	-7 ± 1	0.12	x 0.633 -0.768 0.100<br>y 0.641 0.447 -0.624<br>z 0.434 0.459 0.775	
	Tm^{3+}	12 ± 1	6 ± 1	0.11	x 0.576 -0.805 0.143<br>y 0.660 0.355 -0.662<br>z 0.482 0.476 0.736	
	Yb^{3+}	5 ± 1	3 ± 1	0.15	x 0.593 -0.789 0.163<br>y 0.634 0.333 -0.698<br>z 0.496 0.517 0.698	
V169C	Dy^{3+}	-35 ± 3	-4 ± 2	0.16	x 0.761 0.605 0.234<br>y -0.626 0.780 0.020<br>z -0.170 -0.161 0.972	4.612 0.960 17.233
	Tm^{3+}	31 ± 1	4 ± 1	0.07	x 0.594 0.778 0.205<br>y -0.796 0.606 0.006<br>z -0.119 -0.167 0.979	
	Yb^{3+}	11 ± 1	2 ± 1	0.15	x 0.596 0.763 0.250<br>y -0.796 0.603 0.056<br>z -0.108 -0.233 0.967	
